# Important Flavonoids and Their Role as a Therapeutic Agent

**DOI:** 10.3390/molecules25225243

**Published:** 2020-11-11

**Authors:** Asad Ullah, Sidra Munir, Syed Lal Badshah, Noreen Khan, Lubna Ghani, Benjamin Gabriel Poulson, Abdul-Hamid Emwas, Mariusz Jaremko

**Affiliations:** 1 Department of Chemistry, Islamia College University Peshawar, Peshawar 25120, Pakistan; asad_icp@yahoo.com (A.U.); saries92@gmail.com (S.M.); noreenkhattak777@gmail.com (N.K.); 2 Department of Chemistry, The University of Azad Jammu and Kashmir, Muzaffarabad, Azad Kashmir 13230, Pakistan; lubnaghani41@gmail.com; 3 Division of Biological and Environmental Sciences and Engineering (BESE), King Abdullah University of Science and Technology (KAUST), Thuwal 23955-6900, Saudi Arabia; benjamingabriel.poulson@kaust.edu.sa; 4 Core Labs, King Abdullah University of Science and Technology (KAUST), Thuwal 23955-6900, Saudi Arabia; abdelhamid.emwas@kaust.edu.sa

**Keywords:** flavonoids, polyphenols, quercetin, antioxidative, neuroprotective

## Abstract

Flavonoids are phytochemical compounds present in many plants, fruits, vegetables, and leaves, with potential applications in medicinal chemistry. Flavonoids possess a number of medicinal benefits, including anticancer, antioxidant, anti-inflammatory, and antiviral properties. They also have neuroprotective and cardio-protective effects. These biological activities depend upon the type of flavonoid, its (possible) mode of action, and its bioavailability. These cost-effective medicinal components have significant biological activities, and their effectiveness has been proved for a variety of diseases. The most recent work is focused on their isolation, synthesis of their analogs, and their effects on human health using a variety of techniques and animal models. Thousands of flavonoids have been successfully isolated, and this number increases steadily. We have therefore made an effort to summarize the isolated flavonoids with useful activities in order to gain a better understanding of their effects on human health.

## 1. Introduction

Flavonoids are secondary metabolites, which mainly consists of a benzopyrone ring bearing a phenolic or poly-phenolic groups at different positions [1]. They are most commonly found in fruits, herbs, stems, cereals, nuts, vegetables, flowers and seeds [2,3]. The presence of bioactive phytochemical constituents present in these different plants parts gives them their medicinal value and biological activities [4]. So far, over 10,000 flavonoid compounds have been isolated and identified [5]. Most of the flavonoids are widely accepted as therapeutic agents [6]. These are naturally synthesized through the phenylpropanoid pathway with bioactivity dependent on its absorption mechanism and bioavailability [7].

Flavonoids have been used in natural dyes [8,9], in cosmetics and skin care products [10,11], and anti-wrinkle skin agents [12]. The most pronounced applications of these polyphenols, however, are in the field of medicine. Flavonoids have been used extensively as anticancer, [13] antimicrobial, antiviral, antiangiogenic [14,15], antimalarial, antioxidant, neuroprotective, antitumor, and anti-proliferative agents [16]. Apple peel extracts rich in flavonoids inhibits acetylcholinesterase (ACE) in vitro and is an effective antihypertensive agent [17,18,19,20]. It also prevents cardio-metabolic disorders [21] and displays better preservation of cognitive performance with aging [22].

**Figure 1:**
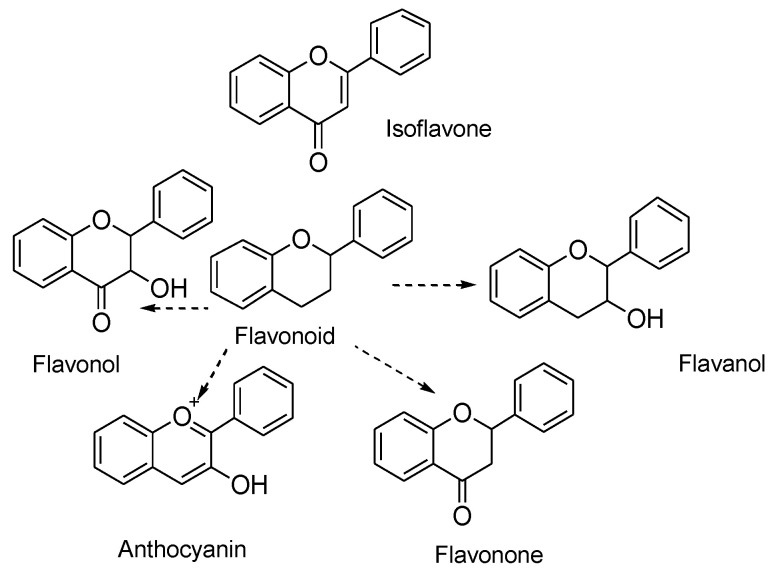
Chemical structure of flavonoids and its different types.

They are classified into various types depending on their chemical structure, degree of unsaturation, and oxidation of carbon ring. Anthoxanthins (flavanone and flavanol), flavanones, flavanonols, flavans, chalchones, anthocyanidins, and isoflavonoids are the different subgroups of flavonoids. Each of these flavonoid is widely distributed in nature (Figure 1) [23]. A higher intake of flavonoid rich foods has a number of health benefits [24]. Since these natural compounds have positive effects on human health, an increasing effort has been made to isolate these compounds from various plants. For instance, citrus fruits are rich sources of flavonoids. Two flavonoids, narigenin and hesperetin, are found in oranges, lemons, and grapes [25]. Anthocyanins and quercetin glycosides flavonoids are present in mulberry [26].

## 2. Effects of Flavonoids on Human Health

### 2.1. Anticancer Action

Cancer is a major health problem caused by abnormal cell growth. There are various anticancer drugs available, and yet only a few display inhibition against oncogenesis, and a majority of them are toxic and have adverse side effects [27]. Natural biomolecules with secondary metabolites have phytomediated content, and display biological activities over a wide range of spectrum, laying the basis for cancer prevention and treatment. Flavonoids are known to inhibit cell growth and act as an anticancer agents [28,29]. Chemoprevention is the use of natural or synthetic substances to inhibit carcinogenesis [30]. Following are copious examples of flavonoids and their use as anticancer agents.

Hesperedin (Hsp) is an important flavonoid which displays efficient anticancer activity [31]. Polylactic-co-glycolic acid (PLGA) nanoparticles were synthesized and loaded with Hsp to form hesperidin nanoparticles (HspNPs) to determine its potential application as an anticancer agent against C6 glioma cells. The encapsulated Hsp exhibited decreased in vitro cell viability against the C6 glioma cell line, and the controlled release of Hsp decreased the cytotoxicity of PLGA [32].

Aurone, a benzo-furanone, is another flavonoid that has been extensively used as an anticancer agent [33]. Various analogues of aurone display different mechanisms against cancer cells because there are many possible targets. These targets include cyclin dependent kinase, histone deacetylase, the adenosine receptor, telomerase, sirtuins, and microtubules [33].

Quercetin is a natural flavonoid present in plants and in commonly consumed foods such as berries, green tea, and grains. It has been used most effectively for colorectal cancer. Cell cycle arrest, increase in apoptosis, antioxidant replication, modulation of estrogen receptors, regulation of signaling pathways, inhibition of metastasis and angiogenesis are among various mechanisms underlying the chemo-preventive effects of quercetin in colorectal cancer [34]. Luteolin, a natural flavonoid with pro-apoptotic activity in hepatocellular carcinoma (HCC) cells, arrests the cancer cell cycle at the G2/M stage. The miR-6809-5p is overexpressed in HCC and it was found to be upregulated by luteolin by directly targeting flotillin-1 [35]. Kaempferol is a natural flavanol, which can reduce the risk of cancer. It stimulates the body’s antioxidants against free radicals that cause cancer [36]. Myricetin is an important flavonoid which has anti-inflammatory and anticancer activities, and in liver cancer it shows antimitotic effects, and it targets different metabolic pathways in mitochondria that result in cancer cell death [37]. *Matricaria recutita* L. (chamomile) flower has a flavonoid content of 157.9 ± 2.22 mg/g QE of dry extract. It displayed dose dependent enhanced mortality of HepG2 cells in HCC. Angiogenesis is an important process which is hijacked for progression of cancer. Vascular endothelial growth factor (VEGF) facilitates blood vessel formation via angiogenesis through VEGF receptors. This expression of VEGF was dose dependently reduced by synthesized extract, making it an efficient anticancer agent [38].

**Figure 2:**
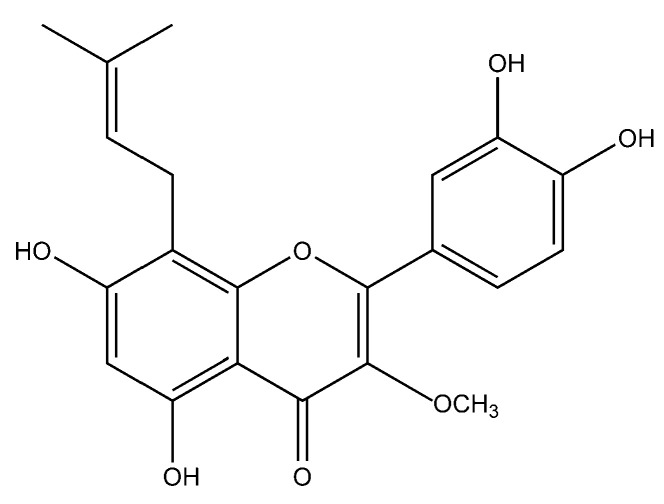
Flavonoid compound isolated from *Sinopodophylli fructus*.

In one of the studies, the aerial parts of *Gastrocotyle hispida* were air dried and used for extraction. The extract showed a flavonoid content of 178 mg/g QE. The in vitro assay of extract was performed for evaluation of its anticancer activity against kidney, liver and breast cancer cell lines [39]. Some reports suggest the flavonoid anticancer activity is because it inhibits protein kinases, which are responsible for regulating cellular pathways [13]. Prenylated chalcone and flavonoids are structurally related and are often considered analogs of each other. Prenylflavonoids were investigated for their potential anticancer activities in vitro [40]. Recently, four prenylated flavonoids isolated from the fruits of *Sinopodophyllum hexandrum* were tested for their cytotoxicity against the human breast cancer cell line T47D. The isolated compounds were spectroscopically analyzed, and structures were elucidated (Figure 2). The percent growth inhibition determined showed an IC_50_ values to be less than 10 μmol·L^−1^ [41]. Apple is rich in many flavonoids which are directly related to improved cardiovascular health [42] and reduces the risk of asthma and Alzheimer’s disease [43]. Flavonoids present in apple are reported to reduce the risk of colorectal cancer. Studies have shown that consumption of one apple per day reduces the chance of cancer up to 50% [44]. Flavonoids extracted from female hop cones have great pharmaceutical importance including anti-carcinogenic and anti-microbial effects [45].

Flavonoids present in *Emblica officinalis* show certain pharmacological activities such as anticancer, antioxidant, anti-inflammatory and as an immunomodulator [46]. Berry flavonoids have a preventive effect on esophageal cancer [47]. The methoxy-flavone also showed cancer chemopreventive properties [48]. Cocoa polyphenols have anticancer effects along with anti-inflammatory and antioxidant activities [49]. Flavonoids extracted from litchi (Epicatechin, proanthocyanidin B2 and proanthocyanidin B4) have shown anti breast cancer activity [50]. Soy isoflavons are active against prostate cancer [51]. Flavonoids found in tea and other flavonoid rich foods such as apple, onion etc., reduce the risk of lung cancers [52].

**Figure 3:**
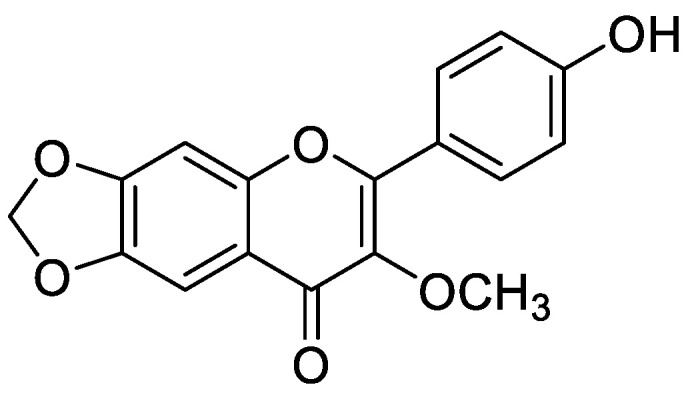
Chemical structure of 4′-hydroxy-6,7-methylenedioxy-3-methoxyflavone.

**Figure 4:**
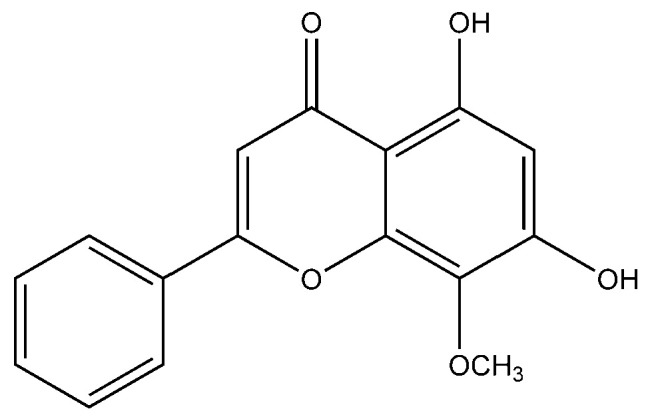
Chemical structure of dihydroxy-8-methoxyflavone (wogonin).

Flavonoid modified drugs (FMD) have much a better effect on lung cancer cell line A549 and L929 [53]. *Allium flavum* and *Allium carinatum* are wild, edible onions rich in flavonoids, rutin, quercetin 3-*O*-glucoside, and kaempferol-3-*O*-glucoside. These, besides having known antioxidant potential, are effective anticancer agents. When combined with doxorubicin, they upregulate angiogenic factor against human hepatoma (HepG2) and lung carcinoma (A549) cell lines [54]. An isolated flavonoid of *Dulacia egleri* showed anticancer activity by inhibiting cathepsins B and L. The extract was prepared from the Dulacia plant, and the compound 4′-Hydroxy-6,7-methylenedioxy-3-methoxyflavone were isolated from it (Figure 3). This isolated flavonoids form the leaves of plant were then spectroscopically analyzed for structure determination. The isolated compound showed enzyme inhibition and can be used for cancer therapy [55]. Licorices (the roots of *Glycyrrhiza uralensis*) contain more than 300 flavonoids, and it is used to alleviate stomach pain, to eliminate phlegm, and to relieve coughing. Recent research proves that it exhibits several pharmacological activities such as antitumor, antiviral and antimicrobial effects [56]. Baicalein and baicalin are phytochemicals which are cytostatic as well as cytotoxic to various human tumor cells, and inhibit tumor growth in vivo [57]. There are a few plants within the genus *Peganum* which have been used as medicines in China for a long time. This genus contains several alkaloids, flavonoids, etc. It has a wide range of biological activities such as antitumor, anticoagulant, anti-parasitic, anti-hypertension and anti-inflammatory properties [58]. Flavonoids and bioflavonoids extracted from *Ouratea* and other genera of the Ochnaceae family have important biological activities such as anti-tumor, antimicrobial, antiviral, and DNA topoisomerase inhibition [59]. One of the most popular Chinese medicinal herbs is *Scutellaria baicalensis*. Wogonin (Figure 4), wogonoside, bicalein, and baicalin are flavonoids extracted from *Scutellaria baicalensis*. These flavonoids are not only cytostatic, but also show cytotoxic effect on tumor cells both in vitro and in vivo [60]. Besides the naturally isolated flavonoids, these can be synthesized in a laboratory using different techniques. Macgown et al. used microwave assisted approach to synthesize novel bisflavonoids, which exhibited anticancer activity against liver, breast and colorectal carcinoma cell lines [61]. Below is an extensive list of plants whose flavonoid content enable them to be used as anticancer agents (Table 1). Although majority of these studies on the anticancer activities of various flavonoids and their derivatives are in pre-clinical study phase, several flavonoids are under consideration for cancer treatment and are at various phases of clinical trials. For example, the icaritin (ICT) is in the third phase of clinical trial for the cure of hepatocellular carcinoma. This prenylated flavonoids lower the signaling process by affecting various receptors like estrogen receptor splice variant ERα36, STAT3 and NFκB, and CXCR4. The ICT also targets ROS pathways and sphingosine kinase-1. It has been suggested to boost the immune response against the developing tumors. The ICT target a number of metabolic pathways, it can be used in glioblastoma and various types of blood leukemia. Due to its promising antitumor properties, various analogs of ICT synthesis and their clinical trials are in progress [62]. Thoracic radiotherapy for lung cancer treatment results in acute radiation-induced esophagitis (ARIE). It has been noted in phase II clinical trials that the epigallocatechin-3-gallatein reduces the ARIE and other related toxicities caused by thoracic radiotherapy [63]. Protein disulfide isomerase (PDI) is an enzyme present in endoplasmic reticulum and normally makes disulfide bonds in newly formed proteins. This enzyme is also involved in tumor progression and neurodegeneration diseases beside other important infectious ailments. The quercetin flavonoid blocks the function of PDI and is a useful anticancer agent. Phase II clinical trials showed that isoquercetin reduce hypercoagulability in cancer patients having high possibility of thrombosis [64]. Further clinical trials should be conducted on flavonoids and their derivatives for anticancer and other diseases as they have lower toxicity and minor side effects.

### 2.2. Antioxidant Activity

Reactive oxygen species (ROS) are produced in the human body mainly as byproducts of the electron transport chain. They are essential for protein phosphorylation, initiation of numerous transcriptional factors, apoptosis, immunity, and differentiation processes. However, ROS also cause oxidative stress upon reacting with molecules such as lipids, proteins, or nucleic acids. Lipid peroxidation by ROS causes cellular membrane damage. This membrane has a potential, with positive charges on the outside of the cell, and negative charges inside the cell. The damage to membrane alters the cell membrane potential and the cell’sosmotic pressure, eventually causing cell death. The human defense system use different mechanisms and enzymes to battle endogenous elevated ROS [76]. Flavonoids act as exogenous antioxidants and are directly oxidized by radicals to form less reactive species via four mechanisms, namely (1) the inhibition of nitric-oxide synthase activity, (2) inhibition of xanthine oxidase activity, (3) modulation of channel pathways, or by (4) interacting with other enzyme systems [77,78].

The antioxidant potential of flavonoids is associated with the molecular structure, and more precisely, with the location and total number of the –OH groups, the conjugation and resonance effects, the surrounding environment which modifies the thermodynamically favored antioxidant site, and the particular antioxidant mechanism for a compound [79,80]. The most commonly used supplemented antioxidants are vitamins C and E. The antioxidant potential of flavonoids is more robust than vitamin C and vitamin E [81]. It is therefore important to regularly include those fruits and vegetables that are rich in flavonoids in daily food intake. For example, due to enhanced and well-known antioxidant and anti-inflammatory properties, flavonoids improve bone health. The utilization of flavonoids in biomaterials has great prospects for bone tissue engineering [82]. This showed that flavonoids should be supplemented in food of aged people. Quercetin, an antioxidant flavonoid, when present in the blood stream, improves vascular health and reduces risk of cardiovascular disease in its conjugated form [83]. The quercetin and its derivatives prevents thrombosis or blood clotting and prevents chances of stroke [83]. Hesperidin and hesperetin are two flavonoids present in citrus fruits and mushrooms that showed antioxidant, anti-inflammatory, antimicrobial and anticancer effects [84]. Propolis has been used as a folk medicine for many years and is now used in the pharmaceutical industry. It contains many compounds, including flavonoids which are responsible for their pharmacological properties such as antioxidant, antimicrobial, healing, and anti-proliferative activities [85]. Rutin, a flavonol, showed a number of biological activities that includes anticancer, antioxidant and cytoprotective etc [86]. Sorghum has a high antioxidant activity in vitro [87]. Flavonoids and tannins from sorghums are the best antioxidants [88]. These results showed that sorghums grains and the products made from these grains should be promoted for food purposes. The sorghum flavonoids have high level of absorption in the small intestine. Although the presence of tannins makes its grains unsuitable for consumption but alkaline cooking reduces the level of tannins by 73% [89,90,91]. It is therefore important to genetically modify the tannin production pathways in sorghum, so that its grains are free from tannins. This crop is also suitable for arid conditions and can grow in harsh environmental conditions. Through this way a flavonoids rich sorghum grains will be available to the human population across the globe. Carotenoids are a class of polyphenols that possess antioxidant activity [92]. Fisetin (3, 3′, 4′, 7–tetrahydroxy flavone) is a flavonoid which shows antioxidant activity along with anti-inflammatory effects [93]. The aerial parts like bulbs, leaves, and flowers of *Eleutherine bulbosa* (Mill.) Urb. have high flavonoid content, and showed antioxidant activity [94]. *Allium cepa*. L (onion) showed antioxidant activity [95]. Dill (*Anethum graveolens* L.) and parsley (*Petroselinum crispum* Mill.) are the medicinal aromatic herbs whose phenolic and flavonoid content made them effective antioxidant agents with the ability to reduce ROS species and prevent ROS mediated diseases such as cancer and cardiovascular ailments. The antioxidant potential of these species may be brought about by different mechanisms and that includes shattering peroxides, and chelating metal ions, which catalyze the oxidation process. This antioxidant activity is related to the phenolic (PC) and flavonoid content (FC); in general, the greater the PC and FC, the greater the antioxidant activity shown by the herbal plant [96]. Utilization of these herbs in daily meals will not only provide aroma and taste but it will improve the nutritional value of food.

*Bryonia alba* L. is a medicinal and homeopathic plant of Cucurbitaceae family with a wide range of biological activities. The methanolic extract of air-dried leaves of this plant was used to isolate four flavonoids (lutonarin, saponarin, isoorientin and isovitexin) using HPLC-DAD. The structures of these compounds were elucidated by NMR and UV spectroscopy, and by mass spectroscopy. Their antioxidant and antiradical activity were confirmed by their ability to suppress ROS produced by macrophages, the isolates being more effective in comparison to the crude extract [97]. The flavonoid and phenolic content, along with the antioxidant potential may vary and depends on the method of extraction used [98]. Leaves extract of piper beetle was prepared using sonication, and had a total flavonoid content of 49.79 ± 1.54 mg QE/gm extract, whereas maceration gave 32.10 ± 0.65 mg QE/gm and Soxhlet extraction gave 40.89 ± 0.87 mg QE/gm (Table 2) [99]. The radical scavenging activity of the leaves extract and its role as an antioxidant agent are closely related to the flavonoid and phenolic content. The aerial part of the *Salvia aristata* was dried and extraction was performed using methanol. The methanolic extract was subjected to the Folin-Ciocalteu and Aluminum colorimetric methods to determine phenolic and flavonoid content, respectively. It turned out that TFC of 50% hydro methanol extract was higher than aqueous and methanolic extract. This was reflected in its potency as an antioxidant agent where IC_50_ of 50% hydro methanol extract was 3.4 whereas aqueous and methanolic extract gave 4.0 and 5.7 (mg/mL), respectively [100]. Plant derived secondary metabolites possess potent antioxidant activity, and this was evident from the ethanolic extract of leaves of *Anisomeles malabarica*. The DPPH assay confirmed its free radical quenching ability to be highest at 120 µg/mL. The extract thus showed enhanced antioxidant and antibacterial properties against *Staphylococcus aureus*, *Bacillus subtilis* and *Proteus vulgaris* [101].

**Figure 5:**
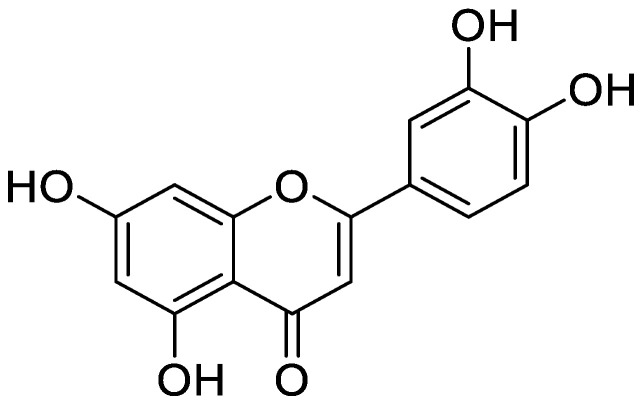
Chemical structure of luteolin.

**Figure 6:**
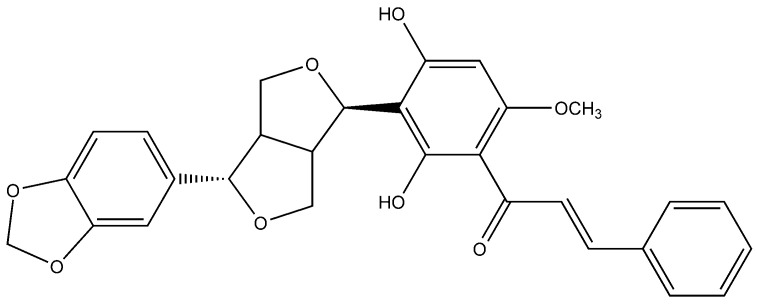
Lead compound of flavonolignan exhibiting antioxidant and antidiabetic activity.

Using natural flavonoids to synthesize its derivatives has immense application and can improve the existing potential of these natural flavonoids. A group of flavonoids derivatives was made through chemically modifying luteolin, apigenin, chrysin, and diosmetin. The synthesis process was carried out by adding different bromo-alkanes with their respective precursors in anhydrous acetone. The derivatives obtained were then assayed for their antioxidant properties using DPPH, FRAP, and ORAC assays. The entire synthesized compounds displayed enhanced antioxidant activity, which may be attributed to their electron or hydrogen radical discharging capacity to DPPH. These derivatives also displayed anti-inflammatory and cytotoxic activity. Luteolin; apigenin; diosmetin; O*^4^*^′^,O*^7^*-dihexyl apigenin, and chrysin halt the function of urease as compared to the rest of flavonoids (Figure 5) [102]. Sesame (*Sesamum indicum*) is used as a starting material and dihydrochalcone, chalcone, flavanone, flavonol, flavone, and flavanol were reacted in a Friedel-Crafts reaction to give rise to a novel flavonolignans series. This one-step process gave twenty different compounds with antioxidant and antidiabetic potential. The α-glucosidase inhibition was investigated using an animal model while the antioxidant activity was determined using DPPH (Figure 6) [103]. The flavonol (−)-(2*R*,3*R*)-5,7-dimethoxy-3′,4′-methylenedioxy-flavan-3-ol isolated from cinnamon activates the nuclear factor erythroid 2-related factor 2 (Nrf2) that has been proven to be an efficient chemical against oxidative stress [104]. In most of the studies on antioxidant potential of flavonoids and its derivatives, only few assays were used. In order to detect the true antioxidant potential of the purified compounds and plant extract, several assays should be conducted so that the effects of solvents, other reagents, and temperature are minimized, and conclusive results are obtained.

**Table 2:** Flavonoids having antioxidant effects.

Plant (Family)—Local Name	Part of Plant	Phytochemical Screening	Total FC	Methods Used Antioxidant Assay	Values of Antioxidant Assay	Bioactivity	Ref.
*Tamarix aphylla* L. (Tamaricaceae)—Athel tamarisk	Leaves	Flavonoid glycosides, carboxylic acid steroids, cardiac glycosides, terpenoids, steroidal compounds, alkaloids, saponins	N/A	DPPH	N/A	Antidiabetic, Hypolipidemic, Antifungal, Antibacterial, Anti-inflammatory, Antioxidant, Wound Healing	[105]
*Oryza sativa* (Poaceae)—Bramo, Serang and Menthi	Caryopsis	Phytosterols, vitamin B group and polyphenols, and polyphenols	N/A	DPPH	(Bramo) 15.25 ± 0.07, (Serang) 25.37 ± 0.07, Menthi (28.15 ± 0.19)	Antioxidant	[106]
*Diospyros kaki*	peel	Vitamins, and flavonoids including catechin, epicatechin, and gallocatechin	N/A	DPPH, FRAP	DPPH (165.75 ± 1.57) FRAP (1609.56 ± 90.88)	Antioxidant	[107]
*Melastoma malabathricum* (Melastomataceae)—karamunting	Leaves and fruits	Terpenoids, phenolic compound, tannin, flavonoids, triterpenes and saponin	N/A	DAPPH	(Leaves) 82% at 50 ppm (Fruit ) 77% at 25 ppm	Antioxidant	[108]
*Rosa damascena* (Rosaceae)—Damask rose	Rose water	Saponins, triterpenoids, tannins, fixed oil flavonoids		Reducing Power Ability (RPA)	3.612	Antioxidant, Skin protecting effect	[109]
*Bauhinia variegate* (Fabaceae)—orchid tree, mountain ebony	Leaves	Anthraquinone, and saponins, erpenoids and alkaloids	11–222.67 mg QE/g	beta carotene bleaching assay.	56.79% inhibition of Beta carotene at 200 ug/mL	Antibacterial, Anticancer, Antioxidant	[110]
*Calotropis procera* (Apocynaceae)—dead sea apple	Roots	N/A	1.62 ± 0.05 mg QE/g	DPPH	42–90%	Antioxidant, metal ion chelating ability	[111]
*Tinospora cordifolia* (Menispermaceae)—heart-leaved moonseed, giloy	Whole plant	Tinocordioside, cordifolide A, palmatine, quercetin, heptacosanol, and syringin	18.91 ± 0.21 mg QE/g	DPPH, MC, FRAP, SA, NO	60–80%	Antibacterial, antifungal, antioxidant, anti-inflammatory activity	[112]
*Vernonia oligocephala* (Asteraceae)—bicoloured-leaved vernonia, groenamarabossie	Roots	flavonoids, saponins, terpenoids, and phenolics	flavonoid 35 (97.35 mg QE/g) contents	DPPH	(% RSC) 90.93 ± 0.66	Antioxidant and inhibitor of AChE, BChE	[113]

### 2.3. Effects on the Cardiovascular System

Dietary flavonoids shows a favorable relationship between their consumption and reduction of cardiovascular diseases [3,114]. Several studies demonstrated that those who consume a large number of flavonoids have 18% lower mortality risk of cardiovascular diseases. Various studies have shown that flavonoids have cardioprotective and neuroprotective [115,116] actions and chemoprotective abilities [117]. Tea is a rich source of flavonoids, and its intake reduces the risk of cardiovascular diseases [118]. Anthocyanidin and proanthocyanidin are flavonoids, that have proven to be effective against cardiac diseases [119]. Isoflavone, anthocyanins [120], and cocoa [121] flavan-3-ols improve vascular health [122]. High consumption of these flavonoids decrease arterial stiffness which reduces the risk of cardiovascular diseases [123]. Oils from leaves and fruits of *Hippophae rhamnoides* (sea buckthorn) have many compounds including flavonoids that showed positive effects on the cardiovascular system [124]. Morin hydrate showed high biological activity such as anti-inflammatory, anticancer, and protection against cardiovascular diseases [125]. Brazil nuts are rich in flavonoids which help to prevent heart disease and cancer [126]. Chrysin is a flavone and has beneficial effects on epilepsy and depression, also suppresses neuro-inflammation, and has neuro-protective effects [127].

For the purpose of pre-clinical studies on animal models, *Primula veris* L. solid herbal extract (PVSHE) effect on myocontractile function was studied [128]. The extract composition of phenolic compounds was investigated by purifying the compounds using column chromatography. The differential UV spectrophotometry showed the total flavonoids concentration. The obtained compounds were then characterized by NMR. An experiment on adult Wister rats was performed in which they were divided into control group, intact group, and experimental group. They were fed with herbal extract of *Primula veris* L. and second experimental group was administered with comparative drug. Cardiodynamic changes were determined using surgical procedures, while myocardial contraction rate was determined computationally. Enzyme linked immune-sorption assay (ELISA) was used for determination of CHF markers. Polymethoxylated flavonoids acted as Primula L. biological markers. The extract was further processed to obtain polymethoxylated flavonoids, flavonoid aglycons and its glycosides along with its constituents. The presence of flavonoids is the most likely reason for decreased ROS production and suppressed peroxide formation, which lead to its cardio-protective effect. A lower number of animal deaths, low CHF markers, and a higher rate of myocardial contraction and relaxation were evident in the results [128]. Several flavonoids have been characterized through NMR spectroscopy. For example, saturation transfer difference (STD)-NMR showed the binding of luteolin and its glycosylated form to the ATP binding domain of multidrug resistant transporter [129]. Thus, these flavonoids have anticancer properties. The antioxidant properties of flavonoids can be accessed through ^13^C-NMR and QSAR methods [130,131].

Morin, a bioflavonoid, has proven to be a cardio-protective agent via animal modeling. Rats were divided into groups, and morin was orally and dose dependently administered to the experimental group. Myocardial necrosis was induced, and the result suggested the improved antioxidant effects and apoptosis. The mechanism for this cardio protection was reported due to alteration of MAPK/NF-kappa B/TNF-alpha pathway (Table 3) [132].

The high flavonoid content of dark chocolate cocoa has proven to be an effective cardioprotective agent [133] by acting as an effective anti-inflammatory agent, by inhibiting NF-kb, by acting as an antihypertensive agent by increasing the bioactivity of nitric oxide, displaying antiatherogenic activity by decreasing triglyceride concentration, and by decreasing insulin resistance and increasing platelet reactivity [134]. Dihydro-quercetin (DHQ), a dihydroxyflavone used in animal models, proved to be effective against cardiac dysfunction by decreasing the generation of ROS and lipid peroxidation and increasing the biological function of antioxidant enzyme. The PI3K/Akt pathway activation is found to have protective effects [135]. Clinical trials on oxerutin showed that it is quite active in treatment of chronic venous hypertension [136]. Interestingly, there are no detected toxicities or side effects of oxerutin clinical trilas results [136]. Different studies and clinical trials showed that a mixture of flavonoids like diosmin, troxerutin, RutinS hesperidin, quercetin, etc., enhance the veins function, soothe the capillary permeability, and increase the lymphatic and hemorrhoidal drainage [137,138]. Thus, flavonoids are useful in control of piles and hemorrhoid diseases. Diosmin that is the flavone glycoside of diosmetin effectively control various types of blood vessel disease like hemorrhoids, bleeding from gums and eyes, etc. [139]. It enhances blood flow inside the body.

**Table 3:** Flavonoids having cardio protective effects.

Plants Whose FCC Have Cardio Protective Effect	Myocardial Injury	Animal/Cell Line Used for Experiment	In-Vivo/Ex-Vivo	Mechanism	*p* Value	Ref.
*Euphorbia humifusa*, *Agrimomia pilosa*, *Juglans regia*	Isoproterenol (ISO)	Male Wistar Rats	In-vivo	activation of PI3K/Akt signaling pathway	*p* < 0.01	[140]
*Dracocephalum moldavica* L (Figure 7, Right side)	Ischemia Reperfusion-induced	Male Sprague-Dawley rats	In-vivo	TFDM halted myocardial apoptosis as mediated by the PI3K/Akt/GSK-3β and ERK1/2 signaling pathways.	*p* >0.05	[141]
Rutin	ischemia-reperfusion (MI/R)	Male Sprague-Dawley rats	In-vivo	SIRT1/Nrf2 signaling pathway is a possible therapeutic target for the treatment of oxidative stress and apoptosis related myocardial diseases	*p* < 0.01	[142]
*Dalbergia stipulacea* and *Hymendictyon excelsum*	N/A	Blood	E-vivo	the extracts produced anti-inflammatory effect due to surface area/volume ratio of cells, and this can be obtained through an extension of membrane or the reduction of the cells volume and an interaction with membrane proteins	*p* < 0.0001	[143]
*Ulva lactuca* (Figure 7, Left side)	cervical decapitation	Hypercholesterolemic mice	In vitro	TNF-a, IL-1b and IL-6 significantly decreased	*p* < 0.05	[144]
*Clinopodium chinense*	Intragastric ISO	Male Sprague-Dawley (SD)	In vivo and In vitro	TFCC safeguard in myocardial injury and increases the cellular antioxidant defense power by stimulating the phosphorylation of AKT, which subsequently triggered the Nrf2/HO-1 signaling pathway	*p* < 0.05	[145]
*Carya cathayensis*	Hypoxia/Reoxygenation	H9c2 cell line	In vitro	Halt the cell apoptosis, which is possibly mediated by changes in the expression of miR-21, PTEN/Akt, and Bcl/Bax.	*p* < 0.01	[146]
*Panax notoginseng*, safflower, *Carthamus tinctorius*	isoproterenol (ISO)-induced MI	Sprague-Dawley rats	In vivo	attenuate the NF-κB signaling pathway, depress the expressions of TNF-α, IL-6, IL-1β, and PLA2	*p* < 0.05	[147]
*Rhododendron simsii*	Myocardial Ischemia/Reperfusion	Sprague-Dawley rat	In vivo	Inhibition of UTR and the further blocking of RhoA/ROCK signaling pathway.	*p* < 0.01	[148]
*Potentilla reptans* roots	ischemia/reperfusion	Male Wistar rats	In vitro	NO release, Nrf2 pathway, and antioxidant activity resulted into lowering of apoptotic index	N/A	[149]
*Gymnema sylvestre* leaves	doxorubicin induced cardiac damage	Male Wistar rats	In vitro	pathological biochemical markers like creatine kinase-MB (CK-MB), lactate dehydrogenase (LDH), serum glutamic oxaloacetic transaminase (SGOT), total cholesterol, triglycerides, uric acid, calcium, nitric oxide and melanoldehyde, and significantly raises the levels of endogenous protective antioxidant proteins	uric acid (*p* < 0.05) total cholesterol <0.05), triglycerides (*p* < 0.05)	[150]

### 2.4. Effects on Nervous System

Flavonoids prevent age related neurodegenerative diseases, and in particular, dementia [151,152], Parkinson’s [153,154,155] and Alzheimer’s disease [156,157,158]. The ROS and nitrogen species (NOCs) have roles in many neurodegenerative diseases. Tangeretin, a flavonoid found in citrus fruits, acts as an antioxidant against ROS and NOCs species and provides protection in neurodegeneration disorders such as Parkinson’s disease [159].

Foods which have abundant amounts of flavonoids lower the hazard of neurodegenerative diseases and also counteract age related cognitive disorders [160]. It is beneficial in two ways; first, it regulates neuronal signal cascade caused by cell apoptosis, and second, shows beneficial effects on the peripheral and central nervous system. Hesperidin (Hsd) and hesperetin (Hst) are two flavonoids known for their neuro-pharmacological effects, including neuroprotective, antidepressant, and effects on memory [161]. Berries contain several natural flavonoids, such as polyphenolic compounds like stilbene, anthocyanins etc. These flavonoids are reported to be effective as anti-neurodegenerative agents, anti-mutagenic and antimicrobial agents [162]. Epicatechin, an antioxidant flavonoid abundantly found in wood plants, has an analog 3-*O*-methyl epicatechin which inhibits neurotoxicity in vitro [163]. The polyphenolic luteolin flavonoid has neuroprotective effects and also as protective effect against age related neuro-disorders [164]. *Forsythia suspensa* is a dried fruit and a Chinese medicinal herb with activities determined against infectious diseases, showing antioxidant activity as well as acting as a neuroprotective agent [165].

Excessive drinking of alcohol causes various health disorders and negatively affects the brain. The acetylpectolinarin (ACP) flavonoid obtained from the *Linaria vulgaris* Mill. has been reported to treat hangover by increasing the spontaneous network function of the cultured hippocampal neurons when treated with low concentration of ethanol. It does so by agonistic action on GABAergic synapses mediated by SK potassium channel [166].

Hyperalgesia is an enhanced pain sensation due to peripheral nerve damage, and is associated with diabetic patients. Quercetin and sodium, when used together, can act as antinociceptives, decreasing diabetes complications [167]. Cisplatin induced hyperalgesia can also be reduced by *6*-methoxyflavone [168].

### 2.5. Prevention of Alzheimer’s Disease (AD)

**Figure 8:**
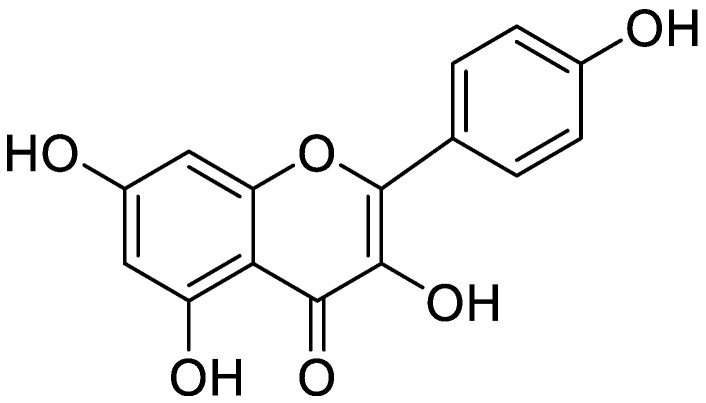
Kaempferol identified in Stachys cretica.

Cyanidin-3-*O*-glucoside, or kuromanin is a flavonoid, a subgroup of anthocyanins that is found in a variety of vegetables and fruits. The ROS are involved in DNA damage, lipid peroxidation, protein and nucleic acid, which resulted into different diseases including Alzheimer’s disease (AD). This can be controlled using natural products, which may slow down its progression by acting as cholinesterase inhibitor, reducing oxidative stress, and preventing neuron damage. This neurodegenerative disorder is identified by excess of amyloid beta (Aβ) fibrils, which accumulates, in the extracellular spaces of brain and tau protein [169]. The neuro decline is thought to be associated with these two proteins. Anthocyanins contain a pseudo aromatic ring C that increases their structural planarity and promotes amyloid fibril disruption due to effective incorporation of anthocyanins inside the amyloid beta fibril groove. They can also cross the membrane separating the blood from the cerebrospinal fluid and prevent neuron degeneration. The compounds of anthocyanin subcategory can therefore be potentially employed as a therapeutic agent in those diseases that are mediated by oxidative stress. Cyanidin-3-*O*-glucoside, an anthocyanin, can therefore act as neuroprotective agent [170]. Similarly, another flavonoid rich plant named gangobilobia may be used in the treatment of age-related dementia and Alzheimer’s disease. Butyrylcholinesterase is an enzyme, found in the blood plasma of humans. Two types of neurotoxic inhibitors of cholinesterase are usually used for AD that are active against acetylcholinesterase (AChE) and butyrylcholinesterase (BChE). Increasing acetylcholine results in better neuron transport and in turn, better cognitive function [171]. Flavonoids have been extensively used as BChE inhibitors (Table 4). The *Hypericum lydium* plant show AChE and BChE inhibition with total FC varying from 4.97 ± 4.56 to 156.44 ± 5.51 mg [172]. The cholinesterase inhibition potential could be linked with a higher number of polyphenolics and flavonoids. Procyanidins, a class of flavonoids, improve cognitive function by means of CREB-SIRT1 [173]. The *Stachys cretica* extracts showed relief of oxidative stress, decrease in Alzheimer’s disease, hyperglycemia, and melisma. A number of phenolic compounds have been identified in the extract of *Stachys cretica* (Table 4 and Figure 8) [174]. The *Actinidia arguta* fruits have six flavonols, seven flavanols, seven phenolic acids, and one anthocyanin with the ability to inhibit AChE and BChE [175]. Most of these extracts and purified flavonoids are active against AD in preclinical studies in murine models that provided very favorable results. It is therefore important to explore these flavonoids in clinical trials so that their usefulness can be shared with the AD patients.

### 2.6. Inhibition of Neuropathy

Nerve malfunction is called neuropathy. Peripheral neuropathy is one of the four types of neuropathy. It refers to the conditions that result when nerves that carry messages to and from the brain and spinal cord and to the rest of the body are damaged or diseased. High levels of glucose destroy the blood vessel that goes to the nerve, and thus, affects the nerves of hand and feet which progress with age. Natural compounds containing flavonoid have been used for to relieve neuropathic pain [184]. A mixture of water and alcoholic solvent has been used for the preparation of root extract of *Cichorium intybus* [185]. Pyridoxine or vitamin B6 is a coenzyme in biological reactions whose high dose intake causes damage to peripheral neurons [185]. This process is known as pyridoxine-induced neuropathy. *Cichorium intybus* is a medicinal plant that contains a variety of beneficial biochemical constituents present, including flavonoid, saponin, and tannin. The presence of these compounds enables them to be used to suppress oxidative stress, and its possible interference of the two amino acid systems, namely GABAergic and glutamatergic systems in nerve injury and neuropathy [186].

Diabetes is associated with a number of complications that includes nephropathy, neuropathy, and cardiovascular disease [187]. A number of studies demonstrate that diabetes neuropathy (DN) and T2DM may be related to increased risk of Alzheimer’s disease [188]. Diabetic neuropathy is a complication that is most commonly faced by 50% diabetes patients, with development of burning sensation to complete loss of sensation of heat and cold in legs and feet, and the loss of peripheral nerve fibers [189]. Adult Sprague-Dawley rats have been used to evaluate the effect of flavanoglycone, hesperidin on DN pain induced by streptozotocin. The animals were divided largely into two groups of diabetic and non-diabetic. After the end of the experiment, the blood serum of used animals were assayed for cholesterol, insulin, etc., determination, while the sacrificed animals were studied for biochemical and molecular investigation immediately after removing the sciatic nerve. Dose dependent reduction in body weight of treated rats were observed at the end of week 8 with *p* < 0.05. The plasma glucose level was observed to be elevated as to that of control group. The combination of insulin and hesperidin decreased the neuropathic pain to a significant amount inducing neuroprotective effect [190]. Nobiletin is a non-polar methoxy flavone found in citrus fruits showing diverse biological activity including anticancer, reverse learning impairment by regulating ERK signal [191], improves memory impairment by reducing AChE expression [192]. Nobiletin has been proven by animal models to dose dependently increase nerve conduction velocity in diabetic ulcer group (DU) [193].

A fat rich diet with a combination of streptozotocin induces glucose intolerance in an animal model; this is then followed by beta cell toxins that are engineered to respond to beta cells. The combination results in sciatic nerve injury, oxidative stress and diabetic neuropathy. The proposed model was used to the effect of flavonoid rich fraction of *Helicteres isora* fruits in diabetic neuropathy in male Sprague-Dawley rats. Thirty rats were divided into two groups, the control and the diabetic neuropathy group. The experiment was performed for three weeks where the diabetic neuropathy group was fed with high fat diet and control group was fed with the pellet diet. After the end of experimentation, the glucose intolerance test confirmed the insulin resistance. This was then followed by dose dependent injection of streptozotocin. Ten days later, the diabetic rats were again grouped into control and FRFHI feed rats. The oral dosage of the extract for further three weeks was continued and then the diabetic neuropathy was assessed by body weight, biochemical parameters and behavioral parameters such as cold, heat, locomotion, and walking test. The FRFHI feed rats restored their body weight, and had reduced blood glucose and cholesterol levels. The FRFHI fed rats also had improved paw and tail withdrawal to heat and cold sensation with improved walking and locomotor activity. The nitric oxide (NO) induced oxidative stress was also noted to be reduced by the extract [194]. Oxaliplatin is a drug that is used in chemotherapy with an adverse side effect of causing painful neuropathy. The compound was introduced into the mice which induced peripheral neuropathy; the flavonoids rutin and quercetin reduced the production of ROS species by acting as an antioxidant agents and reduced the side effect induced by oxaliplatin [195]. The flavonoid quercetin reduced the neuropathic pain by inhibition of *p*-ERK induced in Sprague-Dawley rats by sciatic nerve injury [196].

### 2.7. Stroke Prevention

*Chalcones* are natural precursor compounds of flavonoids and iso-flavonoids. They are present in various plants and vegetables with a wide range of biological activities. Chalcone is an aromatic ketone and an enone with an ability to activate nuclear factor erythroid 2-related factor (2NRF2) pathway. Several novel dihydroxy chalcones were synthesized and evaluated for their ability to suppress ROS species and oxidative stress acting as an anti-ischemic stroke through KEAP1/NRF2/ARE pathway activation. Cerebral ischemia-reperfusion injury (CIRI) in stroke was studied using rat models. These compounds had a potent protection of H_2_O_2_-induced oxidative damage in the neuron-like PC12 cells, but also played a neuroprotective role against ischemia/reperfusion-related brain injury in animals [197].

Ischemic stroke most common therapeutic drug is the tissue plasminogen activator, which due to complications can only be administered to 5% of the patients; therefore, the need to have an effective drug for ischemic stroke is necessary. Different systems such as vascular and inflammatory system interact with each other to form a stable homeostasis of CNS and the semipermeable membrane that separates the circulating blood from the brain and extracellular fluid in the CNS known as the blood brain barrier (BBB). The brain recovers itself from injury using CNS and BBB. The endothelium cells of BBB secretes molecules that adjust the after effect of ischemic stroke (stroke vasculome) when its genes are upregulated [198]. Inflammation after stroke is associated with the upregulation of these inflammation genes. Stem cells can release anti-inflammatory agents. Endothelial progenitor cells (EPCs) directly modulate the inflammation-associated stroke vasculome after ischemic stroke. Fisetin, a flavonoid inhibited LPS-induced TNFα production and suppressing nuclear factor jB activation thus acting as a neuroprotective and anti-inflammatory agent after post ischemia injury in vitro [199]. The cortical development, where neurons in brain failed to migrate in the proper formation in utero, results in malfunctioning known as Focal cortical dysplasia (FCD). The naturally occurring flavonoid rutin has been used on animal models to treat FCD. At a dose of 50 mg/kg, a pronounced recovery was observed in motor neurons. This may act as a clinical drug in the future [200].

### 2.8. *Recovery of Injured Nerves and Anti-inflmmatory Properties*

Injury to the spinal cord or central nervous system (CNS) causes paralysis of the body from the waist down, and this condition is known as Paraplegia. Experimentation of animal model using rats revealed that using isoflurane increased the number of motor neurons. The delayed preconditioning with a neuroprotective effect was observed, which may be associated with the expression of protein complex NF-κB [201]. The *Hypericum perforatum* L. is a medicinal plant with flavonoids as an active phytoconstituent. It has a flavonoid content of 6%. It protects the neuron of adrenal phaeochromo cytoma from oxidative stress caused by ROS species such as H_2_O_2_. Sciatic nerve injury (SNI) was induced in order to determine the effect of plant extract on oxidative stress, cell signaling molecules, cytokine production, and caspase expression in brain muscle. Wistar albino female rats were used for the study. The result suggested a delay in the progression of SNI caused by the plant extract [202]. Flavonoid rich food derived from natural compounds such as mulberry [203], genistein [204], *Acanthus syriacus* [205] etc., display protective effect against sciatic nerve injury.

Angiogenesis is the development of new blood vessels from existing vessels which is important for normal development. Uncontrolled angiogenesis causes serious diseases such as inflammatory disorders, obesity, multiple sclerosis, asthma, endometrioses, and cirrhosis. Plant polyphenols such as flavonoids and chalcones inhibit angiogenesis by regulating multiple signaling pathways [206]. Viscosine is a flavonoid isolated from *Dodonea viscosa*, which has anti-inflammatory, antipyretic and antioxidant properties [207]. Baicalin is a flavonoid present in medicinal plants named *Scutellaria baicalensis* Georgi and *Oroxylum indicum* (Table 5). This flavonoid exhibits antioxidant and anti-inflammatory activity and is used to treat certain diseases such as asthma, liver and kidney diseases, inflammatory bowel diseases, carcinogenesis, and cardiovascular diseases [208,209]. Kaempferol is a flavonoid that possesses anti-inflammatory effects [210]. Rutin is a common dietary flavonoid, having various pharmacological properties such as anti-inflammatory, antimicrobial, and anticancer properties [211]. Chrysin, a type of flavonoid, also has anti-inflammatory and antioxidant effect [212]. It has been observed that fruits which are rich in cyanidin and peonidin have higher anti-inflammatory effects [213]. *Barringtonia racemosa* (L.) leaves and branches water soluble extracts have been used to isolate three acylated flavonoid glycosides which showed moderate anti-inflammatory activity by inhibiting LPS-induced NO production in RAW-2647 cells [214]. The *Eucalyptus globulus* *and Arum palaestinum* herbal extract inhibits interleukin 1 alpha with high phenolic and flavonoid content with potential anti-inflammatory and anti-acne agent for *Acne vulgaris* (Table 5) [215]. Several clinical trials showed that flavonoids have anti-inflammatory properties and block several enzymes involved in inflammation pathways.

### 2.9. Antimalarial Properties

Malaria is caused by the parasitic species of Plasmodium. *Plasmodium falciparum* and other species are now increasingly resistant to the common antimalarial drugs like chloroquine [226], and this resistance is spreading toward artemisinin and its analogues [227,228]. New drugs are required to treat drug resistant strains of Plasmodium [227,228]. In the continuing effort of synthesizing antimalarial drug it is reported that plant extracts that are rich in compounds, such as flavonoids, chalcones, terpenes, quinones, and xanthones, are antimalarial in nature [229]. *Prosopis* is plant genus which has been used for medicinal purposes since ancient times. Its components include flavonoids, tannins, alkaloids etc. These bioactive compounds are antimalarial, antiulcer and antibiotic in nature [230]. Species of the genus *Psiadia* extract contain flavonoids, coumarins, phenylpropanoids, and terpenoids. It shows pharmacological activities such as antimicrobial, antimalarial, antiviral, and anti-inflammatory etc [231]. Extracts of some plants such as *Psiadia dentata* and *Psiadia arguta* inhibit the growth of *Plasmodium falciparum* [231]. *Waltheria indica* (*syn. Waltheria americana*) extract have flavonoids, (−) epicatechin, kaempferol, quercetin, sterols etc. It is used for the treatment of malaria and other infectious diseases (e.g diarrhea due to *Escherichia coli*, lungs infection due to *Klebsiella pneumoniae*), inflammation, and prevention of oxidative stress [232]. Prenylated flavonoids obtained from the bark of *Artocarpus styracifolius* show antiplasmodial and antitrypanosomal effects [233]. Silymarin from *Silybum marianum* has been isolated and purified. Anti-plasmodial activity of silymarin, a polyphenolic flavonoid, has been reported and it forms the silymarin-heme complex, and inhibits the conversion of toxic free heme into crystalline non-toxic heamozoid [234]. *Indigofera oblongifolia* leaf extract has significant effects against malaria and protects the liver from injury caused by *P. chabaudi* via antioxidant and anti-inflammatory ways [235].

### 2.10. Antiviral Activities

**Figure 9:**
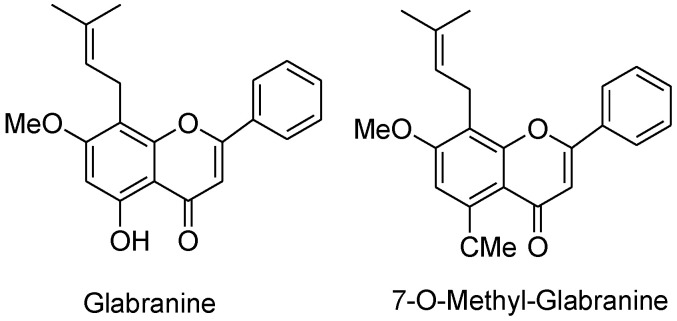
Chemical structures of glabranine and 7-*O*-methyl-glabranine.

Patients with weak immunity when infected with a contagious viral infection can be deadly and the recent novel coronavirus pandemic showed that immunocompromised people are especially vulnerable to the COVID-19 [236]. Serious efforts have been made to synthesize antiviral agents with efficient activity. Natural bioactive flavonoids present in medicinal plants and herbs have been widely reported to have antiviral activity and are concentrated and modified for its better action [237]. *Houttuynia cordata* Thunb is a plant found in Eastern Asia, having promising antiviral activities against enveloped viruses such as influenza virus, herpes simplex virus-1 and human immunodeficiency virus-1 in vitro [238]. Both natural and synthetic flavonoids are potential medicines for many diseases, including HIV [239]. Glabranine and 7-*O*-methyl-glabranine are the two flavonoids extracted from Mexican plant *Tephrosia madrensis*. The plaque assay confirmed that these isolates exhibit antiviral activities, and inhibit dengue virus replication. The structures of the isolates were determined spectroscopically and then the stoichiometry was analyzed alongside cytotoxicity using [3*H*]-thymidine assay. At a concentration of 25 μM, glabranine inhibited the dengue virus by 76.9% while 7-*O*-methyl-glabranine inhibited the replication of virus at the same concentration by 75%. Both of these compounds have a prenyl side-chain in C-8 which is considered to have a role in inhibition of virus (Figure 9) [240], and catechins against the influenza virus [241]. Anthocyanins found in berry fruits have antiviral activity against influenza virus [242]. Baicalein, quercetin, and fisetin are active inhibitors of chikungunya virus [243]. The epicatechin 3-gallate, fisetin, quercetin reduces murine norovirus and daidzein, and kaempferol reduce feline calicivirus [244]. Four flavonoids (5-hydroxy-7,8-dimethoxyflavone, 5-hydroxy-6,7-dimethoxyflavone, acacetin, apigenin) extracted from *Mosla scabra* display antiviral activity against influenza viruses [245]. Burs (involucre) of *Castanea crenata*, have a wide range of biological activities attributed to the bioactive phtyo-constituents present in it that includes flavonoid, tannins, phenolic acids, coumarins, phenylpropanoids, and steroids. The antiviral activity of the extract was determined by SRB method using cytopathic effect (CPE) reduction assay in HeLa or Vero cells. The isolated flavonoid, kaempferol, showed enhanced antiviral properties against HRV1B, CVB3, and PR8 [246]. Using molecular docking and simulation studies, flavonoid analogues are also found to be active against H1N1 virus neuraminidase [247]. Of the various types of influenza virus, IAV is a single stranded RNA virus that is dangerous and most responsible for an annual deaths of around 500,000 globally. The antigenic shift give rise to new a subtype of viruses, this occurs when two or more strains of same virus or of different viruses combine or mutate to form a new subtype that is resistance to available, common antiviral agents. Purified guava flavonoid glycosides inhibit IAV replication by early regulation of interleukin-1 beta and interleukin-8 via protein gene expression [248].

*Scutellaria baicalensis* of Lamiaceae family has a beneficial health impacts as its roots contain flavonoid as bioactive component, and has been used for centuries as medicinal herb. Using an animal model, influenza virus was introduced through the nose, and after two hours, the root extract was administered. The histopathology indicated the reduction of oxidative stress by inhibiting NO production as well as significant inhibition of IAV-infected mice with an increased survival rate [249].

### 2.11. Antibacterial Action

**Figure 10:**
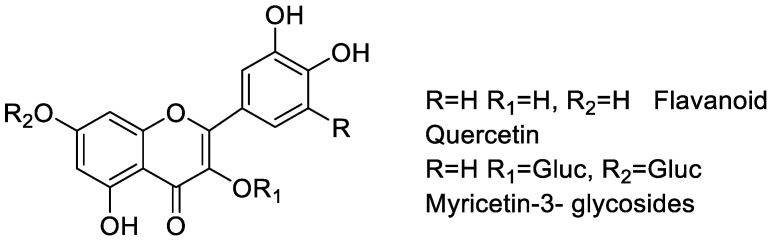
Flavonoids of Bridelia extract.

The increase and spread of multi-drug resistance in pathogenic bacteria resulted into a number of antibiotics that have become ineffective for the treatment of a number of bacterial infections [250,251]. Flavonoids have the ability to enhance the protective immune systems of humans [78]. Several flavonoids act both as bacteriostatic and bactericidal agents by damaging the cytoplasmic membrane, and inhibiting energy metabolism and nucleic acid synthesis of microorganisms [252]. *Bridelia* is a plant of genus of Phyllanthaceae family, which is used as a pain-relieving medicine in Asia and South Africa. Its extract contains flavonoids like quercetin, gallocatechin-(4′-*O*-7)-epigallocatechin, myricetin-3-glycosides, and isoflavone. These flavonoids are responsible for its antimalarial, antibacterial, anti-inflammatory activities (Figure 10) [253]. Anthocyanidin plays an important role against tuberculosis and drug-resistant *Mycobacterium tuberculosis* strains [254]. Studies have shown that hesperidin and hesperetin, two flavonoids, show very good antimicrobial activity [255]. Flavonoids from *Cuscuta* are reported for many biological activities, including antiproliferative, hepatoprotective, anxiolytic and antimicrobial activities [256]. *Turbina corymbose* is a natural source of antimicrobial and tyrosinase inhibitor [257,258]. There has been extensive literature available on flavonoid rich plants showing antibacterial activities, such as flowers from *Acacia saligna* (Labill.), roots and aerial extract of *Lamium album* [259,260], *Tridax procumbens*, [261], Tunisian Date Palm seeds [262], Aurone derivatives [263], Chrysoeriol [264], *Alanchoe mortagei*, *K*. *fedtschenkoi* [265], Onion (*Allium cepa* L.), *Asplenium nidus* (fern) [266], *Trianthema decandra* [267], pineapple (*Ananas comosus*) [268], *Pseudarthria hookeri* (Fabaceae) [269], Keigairengyoto [270], *Quercus brantii* L. [271], *Acacia saligna* (Labill.) [260], or the use of flavonoid rich marine algae such as marine algae *Sargassum swartzii* [272], all of which show significant antimicrobial activities.

### 2.12. Antidiabetic Effects

It is found that cranberry flavonoids decrease the blood glucose levels and increase insulin sensitivity in animals [273]. Anthocyanins control obesity and consequently can also help in the prevention of type 2 diabetes [274]. Chrysin is natural flavone having many health benefits such as antidiabetic, antiallergic, anticancer, and antitumor effects [275]. Many flavonoids are anti-diabetic by increasing secretion of insulin, improvement of hyperglycemia, reduce resistance to insulin and increase uptake of glucose by skeletal muscles in murine model [276]. Flavonol rich chocolate enhances insulin sensitivity and reduce insulin resistance in healthy subjects [277]. *Allium cepa* L. (onion) flesh and skin [278] are rich in quercetin derivatives [279] and display protein tyrosine phosphatase 1B (PTP1B) inhibitory activity, decreases its expression, and increases glucose uptake making it a potential antioxidant and antidiabetic agent [280].

Flavonoids have many important functions in gastrointestinal tract i.e glucose homeostasis and lipid metabolism [281]. Anthocyanins obtained from black beans have antidiabetic potential [282]. *Melicope lunuankenda* leaves extract have O-prenylated flavonoids which shows antidiabetic activity against type 2 diabetes [283]. Flavonoids in black carrots (*Daucus carota*) are therapeutic agents against obesity and diabetes mellitus [284]. Naringenin and its glycosides found in citrus fruits has antioxidant and anti-diabetic activity [285]. The genus *Sterculia* have many natural products compounds including flavonoids and polyphenols which have antidiabetic and antimicrobial activities [286].

Kaempferol, a natural flavonoid, acts as antidiabetic agent by inhibiting cell proliferation, decreases PI3K, P63, SREBP-1 expression and phosphorylates insulin resistance substrates [287].

*Warionia saharae* is a flavonoid rich plant. Its extract was used on animal model to prove its antidiabetic properties. Treptozotocin (STZ) induced diabetic rats were used as test subject and normal rats were used as control group. Oral administration of the extract was continued for 15 days and its histopathological examination of liver along with glucose tolerance test was performed. The state of the liver and pancreas was found to be improved with the test group rats and had the potential to act as an antioxidant as well as an efficient antidiabetic agent [288]. In order to overcome the absorption and bioavailability issues of flavonoids, novel strategies should be developed for their maximum absorption. In one such study, baicalin, which is hydrophilic and poorly absorbed glycosylated flavonoid, was loaded inside nano-structured lipid carriers [289]. These baicalin containing a lipid carrier were tested in diabetic rats and its was observed that they have significant antidiabetic efficacy by halting the lipid peroxidation [289].

Although there are several studies in murine models that showed that, the hesperidin controls the blood glucose level, clinical trials in human showed no such observation [290]. Thus majority of clinical studies hints that hesperidin did not modulate insulin or enzymes of the glucose metabolic pathway. There are some limitation of these clinical trials related to absorption of hesperidin [290].

Methylglyoxal is the metabolite whose concentration increases in the blood during diabetes and causes atherogenesis (a condition where artheromatous plaque develop in arteries, with blockage problems) and it also attaches with nerve ending resulting in nerve damage [91]. Clinical trials on quercetin indicated a decrease in the plasma level of methylglyoxal while epicatechin has no promising effect. This showed the different potency of various flavonoids against specific disease.

### 2.13. Antifungal Properties

**Figure 11:**
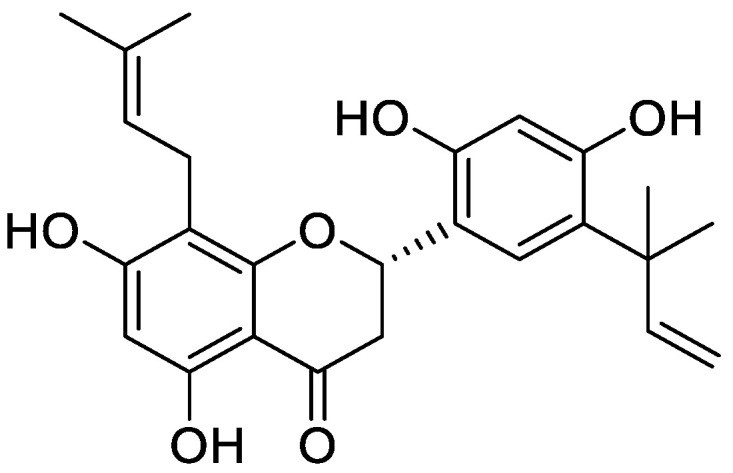
Chemical structure of 2′,4′-dihydroxy-5′-(1‴,1‴-dimethylallyl)-8-prenyl pinocembrin (8PP).

New antifungal agents are required as the currently available antifungal drugs are not effective completely due the development of resistance and undesirable side effects [291]. Leaves of *Aquilaria* have many bioactive compounds including flavonoids responsible for its antiviral, antifungal, and antitumor activities [292]. *Artemisia sacrorum* extract have two flavonoids namely sacriflavone A and sacriflavone B and both of them have antifungal potency [293]. Moreover, 2′,4′-dihydroxy-5′-(1‴,1‴-dimethylallyl)-8-prenylpinocembrin (8PP) is a natural prenyl flavonoid isolated from *Dalea elegans*, which shows antifungal effects against *Candida albicans* biofilms (Figure 11) [294].

## 3. Conclusions

Flavonoids are groups of various compounds found naturally in many plants, such as fruits and vegetables, along with plant products such as coffee, chocolate, and tea. It had been repeatedly reported that flavonoids possess a wide range of health benefits. For example, flavonoids are rich in antioxidants, providing our body with natural immune protections from daily environmental and endogenous toxins. Different classes of flavonoids are so far isolated with several significant biological activities such as anticancer, antibacterial, antifungal, anti-diabetic, antimalarial, neuroprotective, cardio-protective, anti-inflammatory. Thus, including different types of flavonoids in daily diet is highly recommended to stay healthy and to reduce the risk of serval life threatening diseases such as diabetes mellitus, cancer as well as lowering the risk of having stroke and heart attack. The therapeutic effects of flavonoids have been proved in majority of pre-clinical studies in murine models. Different approaches should be used in clinical trials so that the absorption and bioavailability of flavonoids are not compromised. Their production should be increased through expression of their biosynthetic pathway enzymes in other plants and species with rapid growth. The funding agencies should facilitate research on flavonoids due to their versatile role in health and wellness. Further, the conjugates of flavonoids with other important drugs may enhance the potency of those compounds. Decisively, more research work is needed to resolve more the structures opf more flavonoids and to investigate their therapeutic applications. Flavonoids structures will always inspire research for the design and synthesis of new effective drugs for different types of diseases.

## Figures and Tables

**Figure 7:**
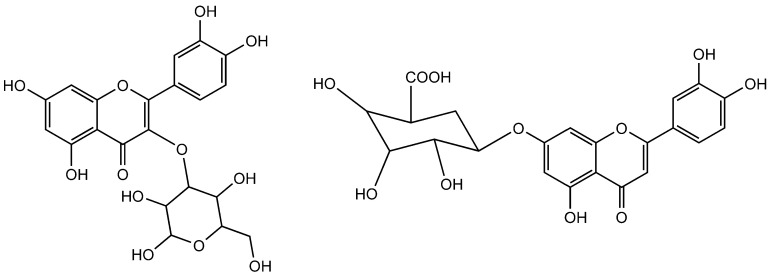
Quercetin-3-*O*-glucoside (Left side) and luteolin-7-*O*-β-d-glucuronide (Right side).

**Table 1:** Flavonoids with anticancer properties.

Isolated from	Isolated Flavonoids	Total Flavonoid Content	Type of Cancer	Mechanism of Action	Anticancer Assay	Ref.
Milk thistle (*Silybum marianum*)	Silybin or silibinin	N/A	cervical (HeLa) and hepatoma (Hep3B) human cancer cells	Inhibits hypoxia-inducible factor-1a and mTOR/p70S6K/4E-BP1 signaling pathway	N/A	[65]
Silybin	Modified Flavonoids silybin derivatives namely 2,3-dehydrosilybin (DHS), 7-*O*-methylsilybin (7OM), 7-Ogalloylsilybin (7OG), 7,23-disulphatesilybin (DSS), 7-*O*-palmitoylsilybin (7OP), and 23-*O*-palmitoylsilybin (23OP)	N/A	human bladder cancer HTB9, colon cancer HCT116 and prostate carcinoma PC3 cells	silybin strongly synergizes human prostate carcinoma cells to doxorubicin-, cisplatin-, carboplatin-, and mitoxantrone-induced growth inhibition and apoptotic death	N/A	[66]
*Silybum marianum*	Silybin nanosuspension	N/A	human prostatic carcinoma PC-3 cell line	silybin nanosuspension induced PC-3 cell growth inhibition and Silybin nanosuspension-induced apoptosis may occur in the G1 phase.	N/A	[67]
*Silybum marianum*	Silibinin	N/A	MCF-7 breast cancer cells.	blocks rapamycin signaling with a concomitant reduction in translation initiation	N/A	[68]
*Cnidoscolus quercifolius*	N/A	N/A	prostate (PC3 and PC3-M) and breast (MCF-7) cancer cells	N/A	N/A	[69]
*Lasiosiphon eriocephalus*	N/A	N/A	HeLa and MCF-7	N/A	N/A	[70]
Onions, Kale, French beans, lettuce etc	Quercetin	N/A	ovarian PA-1 cancer cell line	quercetin induces the mitochondrial-mediated apoptotic pathway, and thus, it inhibits the growth of 17 metastatic ovarian cancer cells	N/A	[71]
*Stachys tmolea*	Erbascoside, chlorogenic acid, andapigenin7-glucoside	Total flavonoids (mg QEs/g dry plant) 4.98 ± 0.06	N/A	N/A	N/A	[72]
*Cassia occidentalis*, *Callistemon viminalis*, *Cleome viscosa* and *Mimosa hamata*	N/A	*C. viminalis* (46.41 ± 2.23 mg of CAE/g DW) and *M. hamata* (40.33 ± 1.16 mg of CAE/g DW) followed by *C. viscosa* leaves (36.22 ± 0.74 mg of CAE/g DW), *C. occidentalis* (35.32 ± 0.70 mg of CAE/g DW) and *C. viscosa* root (33.63 ± 1.25 mg of CAE/g DW)	human breast cancer cell line MCF-7	anti-angiogenic activity via inhibition of blood constituents density in vessels	SRB assay	[73]
*Brassica oleracea* var. *alboglabra*	N/A	N/A	human cancer cell lines (colon cancer cell line SW480, liver cancer cell line HepG2, cervical cancer line HeLa, and lung cancer line A549	N/A	MTT assay	[74]
*Melodorum siamensis*	N/A	N/A	pancreatic β cell line MIN-6 cells	Nuclear factor-κB inhibition	N/A	[75]

**Table 4:** Flavonoids with Anti-Alzheimer’s effect.

Type of Flavonoid	Inhibition	Lead Compound	Mechanism	Ref.
Galangin, kaempferol, quercetin, myricetin, fisetin, apigenin, luteolin and rutin	BChE	Galangin	Docking study showed that flavonoids bind to the BChE active site by forming multiple hydrogen bonds and π-π interactions.	[176]
7-Aminoalkyl-Substituted Flavonoid Derivatives	AChE and BChE	2-(naphthalen-1-yl)-7-(8-(pyrrolidin-1-yl) octyloxy)-4*H*-chromen-4-one	Compound targeted Catalytic active site (CAS) and the peripheral anionic site (PAS) of AChE	[177]
*Plectranthus scutellarioides* flavonoids	AChE and BChE	flavonoids apigenin 7-*O*-(3′′-*O*-acetyl)-β-d-glucuronide, apigenin 5-*O*-(3′′-*O*-acetyl)-β-d-glucuronide	N/A	[178]
*Salvia hispanica*	AChE and BChE	Colored chia seeds	Rich in polyphenols, quercetin and 23 isoquercetin with a positive correlation with inhibition of ChEs activity	[179]
*Nardostachys jatamansi*	AChE and BChE	Leaves and rhizome of plant extracts	Presence of phytochemicals such as flavonoid and phenols	[180]
*Leiotulus dasyanthus*	AChE and BChE	pimpinellin (66.55%) and umbelliferone (40.99%)	N/A	[181]
Arceuthobium	AChE and BChE	Ethanolic Plant extract	Higher flavonoid phenol content exhibited higher inhibition by protecting the brain against oxidative stress	[182]
Salvia (sage) species	cholinesterase inhibition	Dichloromethane and ethanol extracts of the aerial parts of Salvia cryptantha	Strong inhibitory activity of the CH_2_Cl_2_ extract of aerial parts of *S. cryptantha* could also be presumed to emerge from its terpene content and synergic type interaction	[183]
Woundwort plants (Stachys species) flavonoids	AChE (MeOH), BChE inhibitory (EtOAc)	*Stachys cretica*	Apigenin, Hesperidin and Kaempferol have a positive correlation with inhibition of AChE and BChE	[174]

**Table 5:** Anti-inflammatory activity of Flavonoid’s.

Plant (Family)—Local Name	Part of Plant	Phytochemical Constituents	Isolated Compounds	Assay	Flavonoid Inhibition	Mechanism	Biological Activity	Ref.
Lotus plumule (Nymphaeaceae)	Fresh plant	Alkaloids and flavonoids, polysaccharides, tannins, proteins and fats	N/A	Cell viability assay, Griess reagent protocol, enzyme-linked immunosorbent assay	N/A	Inhibit the production of NO radicals, PGE2 and TNF-α and pro-inflammatory cytokines IL-1β and IL-6	Antioxidant and anti-inflammatory	[216]
*Cirsium japonicum* (Asteraceae)	Dried powder	Phenolic acids, lignans, polyacetylenes, polysaccharide, sterols, triterpenes, sesquiterpene lactones, and alkaloids	flavonoids, saponins, polysaccharides, essential oil, coumarin and alkaloids	Nitric oxide (NO) and IL-6 measurement Quantitative real-time PCR analysis, Western blot	Flavonoids 94.2% NO inhibition	Flavonoids, saponin and essential oil inhibit NO production	Anti-inflammation, anti-cancer and anti-atherosclerosis	[217]
Lychee (*Litchi chinensis* Sonn.) (Sapindaceae)	Dried Seeds	oligosaccharides, phenolics, flavonoids	fifteen flavonoids	NO inhibitory assay	IC_50_ of Extracted flavonoid 43.56 ± 2.17 μM	N/A	Anti-inflammatory and antioxidant	[218]
*Dillenia suffruticosa*- simpoh air (Dilleniaceae)	Fresh leaves	triterpenoids, flavonoids, and their glycosides, the anthraquinone glycosides, phenolic derivatives, and tannins	triterpenoids betulinic acid, koetjapic acid, flavonoids vitexin, tiliroside, kaempferol	In vivo rat model of acute λ-carrageenan-induced paw oedema	Vitexin (27.97 ± 0.01% inhibition of COX-1 and 45.35 ± 0.01 of COX-2 at 200 μg/mL), (kaempferol) 9.89 ± 0.02 COX1 ± COX-2 49.25 ± 0.02, (tiliroside) COX-1 19.79 ± 0.00, COX-2 37.59 ± 0.01	potent inhibition of COX-2 than COX-1 reaction	Anti-inflammation	[219]
naringenin, naringenin chalcone, and quercetin				arachidonic acid-(AA) and tetradecanoylphorbol-13-acetate-(TPA) induced ear edema			anti-inflammatory and antiallergic activity	[220]
*Severinia buxifolia* (Rutaceae)	Branches	acridone alkaloids, tetranorterpenoids, coumarins, limonoids, and sesquiterpenes	N/A	albumin denaturation, membrane stabilization, and antiproteinase activity	The *S. buxifolia* methanolic extracts IC_50_ value against albumin denaturation was (μg/mL) 28.86 ± 4.80	It is possible that bioactive compounds in the extract protect lysosomal membranes activation of phospholipases. for the anti-inflammatory activity of *S. buxifolia* extracts via a membrane stabilization effect	Antioxidant, anti-inflammatory	[221]
*Scutellaria moniliorrhiza* (Lamiaceae)	Herb	N/A	Four flavonoid compounds	Bioassay using rats	Inhibitory activities with IC_50_ values being in the range of 2.29 e3.03 mM.	N/A	anti-inflammatory activities, inhibitory activities against aldose reductase	[222]
*Citrus reticulata*—*Orange*	Dried peel	Flavonoids, Phenolic acids	N/A	Levels of iNOS and COX-2 mRNA in RAW 264.7 cells were measured using RT-PCR	N/A	highest content of nobiletin and tangeretin, also produced a strong affinity to inhibit iNOS and COX-2 expression in LPS and IFN-c induced Raw 264.7 cells. We attribute this observation to the presence of a greater number of methoxy groups in nobiletin compared to the other flavonoid species studied.	antioxidant and anti-inflammatory	[223]
Black mulberry (*Morus nigra* L.) (Moraceae)	Fruit	N/A	N/A	ELISA to detect the pro-inflammatory cytokines IL-1β, TNF-α, IFN-γ, and NO in the serum of mice	*Ear edema* 65.2% inhibited	inhibitory activities of proinflammatory cytokines	Antinociceptive, Anti-inflammatory	[224]
*Citrus bergamia*—bergamot (Rutaceae)	Juice	Neohesperidin, naringin, melitidin, neoeriocitrin, hesperetin, naringenin		N/A	N/A	Inhibit intestinal inflammation by reducing: ROS/RNS production—inflammatory NF-κB and MAPKs pathways—pro-inflammatory cytokines levels and neutrophil infiltration—adhesion molecules expression—oxidative and nitrosative stress—tissue injury	Anti-inflammatory and antioxidant activities	[225]
